# Supplementary material for: Phylogenetic Analysis of the PR-4 Gene Family in Euphorbiaceae and Its Expression Profiles in Tung Tree (Vernicia fordii)
Source: Plants (Basel). 2023 Sep 1;12(17):3154. doi: 10.3390/plants12173154 (PMC10490464; doi:10.3390/plants12173154)
Supplement: Supplementary file 1 [file plants-12-03154-s001.zip › plants-2480902-supplementary/Supplymentary Table1.pdf]

Supplymentary Table 1 Primer of PR-4 genes in tung tree

Primer name	Primer sequence (5'→3')	Tm value (℃)	Primer function
VF16136-PR4-dlF1	TGAGCAATGTGGTAGGCAAG	60	Quantitative real-time PCR
VF16136-PR4-dlR1	ACTGCATTCAGATCCCATCC		
VF16135-PR4-dlF1	GCACAAATTGGATGGGACTT	60	Quantitative real-time PCR
VF16135-PR4-dlR1	GTAAGCAACTGCCACAAGCA		
VF16136-F	ATGGGAAGAGTTATTAAAATATGTAT	51	Gene clone
VF16136-R	TTACTGATCATCTATAATAGAAAGCA		
VF16135-F	ATGGGAAGGGTAATTAGCATAA	51.5	Gene clone
VF16135-R	TTAATCACCACAATTAACAAACTG		
VF16136-1300-F	AAGAGACAGGATCCGAATTCATGGGAAGAGTTATTAAAAT	56	Subcellular location analysis
VF16136-1300-R	ATCGGTGCACTAGTGTCGACCTGATCATCTATAATAGAAA		
VF16135-1300-F	AAGAGACAGGATCCGAATTCATGGGAAGGGTAATTAGCAT	56	Subcellular location analysis
VF16135-1300-R	ATCGGTGCACTAGTGTCGACATCACCACAATTAACAAACT		
JQ680036_EF1α	GCCTGGTATGGTTGTGACCT	60	Reference gene
JQ680036_EF1α	GGATCATCCTTGGAGTTGGA		
