# Supplementary material for: Phylogenetic Analysis of the PR-4 Gene Family in Euphorbiaceae and Its Expression Profiles in Tung Tree (Vernicia fordii)
Source: Plants (Basel). 2023 Sep 1;12(17):3154. doi: 10.3390/plants12173154 (PMC10490464; doi:10.3390/plants12173154)
Supplement: Supplementary file 1 [file plants-12-03154-s001.zip › plants-2480902-supplementary/Supplymentary Table2.pdf]

>VF16135

MGRVIKICIVVLLCLVGGSVAEQCGRQAGGKLCPNNLCCSQFGWCGSSDDYCSPSKNCQS
NCEGGGGGGGQSASNVRATYHLYNPQQHGWDLNAVSAYCSTWDANKPYSWRSKYGWTAFC
GPVGPRGQASCGKCLRVTNTRTGAQTTVRIVDQCSNGGLDLDVNVFRKLDTDGKGYQQGH
LTVNYQFVNCGDSFNPLLSIIDDQ

>VF16136

MGRVISIILCTVFLVFLIVRANSQSASNVRATYNPYNPAQIGWDLNTASVFCATWDAGKP
LEWRQKYGWTAFCGPVGPQGQDACGSCLLVTNTGTGAQVTVRIVDQCSNGGLDLEEGVFR
QIDTDGKGIAQGHLIVNYQFVNCGD
